# Psychological depression and its association with oocyte yield and embryo outcomes in infertile women undergoing *in vitro* fertilization-embryo transfer

**DOI:** 10.3389/fpsyt.2026.1796497

**Published:** 2026-05-15

**Authors:** Xiao-huan Song, Yi-tao Yin, Yue-di Jia, Jie-yu Wang, Heng Wang, Dan-ni Wang

**Affiliations:** 1 School of Public Health, Anhui Medical University, Hefei, Anhui, China; 2 The First Clinical Medical College, Anhui Medical University, Hefei, Anhui, China; 3 Reproductive Medicine Center, Department of Obstetrics and Gynecology, The First Affiliated Hospital of Anhui Medical University, Hefei, Anhui, China; 4 The First Affiliated Hospital of Anhui Medical University, Hefei, Anhui, China; 5 Oxford University’s Nuffield Department of Primary Care Health Sciences, University of Oxford, Oxford, United Kingdom

**Keywords:** depression, infertility, IVF-ET, pregnancy outcome, transferable embryo

## Abstract

**Background:**

Women undergoing *in vitro* fertilization–embryo transfer (IVF-ET) for infertility are at increased risk of depression; However, the association between depressive symptoms and intermediate outcomes during assisted reproductive treatment remains unclear. This study aimed to investigate the association between depressive symptoms and IVF-related intermediate reproductive outcomes.

**Methods:**

A total of 183 infertile women undergoing IVF-ET at the Reproductive Medicine Center of the First Affiliated Hospital of Anhui Medical University were enrolled during 2021. Depressive symptoms were assessed prior to treatment initiation using the Patient Health Questionnaire-9 (PHQ-9). Clinical data related to oocyte retrieval and embryo development were collected after completion of the IVF cycle. Multivariable linear regression models were used to examine the associations between depressive symptoms and reproductive outcomes. Restricted cubic spline (RCS) models were applied to explore dose–response relationships.

**Results:**

The mean age of participants was 30.80 years, and 18.1% met the criteria for moderate or severe depressive symptoms. After adjustment for potential confounders, higher PHQ-9 scores were associated with a reduced number of transferable embryos (β = −2.17, 95% CI: −3.64, −0.69), while the association with retrieved oocyte number was of borderline statistical significance (β = −3.02, 95% CI: −6.05, −0.002). RCS analyses demonstrated a negative dose–response association between depression severity and IVF-related intermediate reproductive outcomes, with no evidence of nonlinearity (*P* nonlinear > 0.05).

**Conclusion:**

Depressive symptoms among infertile women undergoing IVF-ET are significantly associated with a reduced number of transferable embryos, suggesting that mental health status may play an important role in intermediate assisted reproductive outcomes.

## 1 Introduction

Infertility is a state of low fertility in which a couple has not used contraception and has had regular sex for at least 12 months without achieving a clinical pregnancy (1). It has become a prevalent global health concern and is recognized by the World Health Organization as a major public health issue (2). For families, numerous studies have shown that infertility can affect a couple’s normal physical, emotional, and social well-being (3, 4). Due to the effects of the disease itself and the physical, psychological, and financial stresses associated with the choice of *in vitro* fertilization-embryo transfer (IVF-ET) treatment, it is easier to experience negative emotions such as anxiety and depression, resulting in a high prevalence of psychopathology in infertile couples undergoing IVF-ET treatment (5, 6). Women tend to be more psychologically vulnerable to stressors and mood disorders than men, and women with infertility are more likely to experience anxiety and depression than men (7, 8).

Assisted reproductive technology (ART) treatment is currently an effective method for resolving infertility and has been widely accepted by infertile couples (9). Nevertheless, despite advances in ART, the live birth rate following one complete IVF cycle remains relatively modest, at approximately 29.1% (10), so finding innovative ways to improve the success rate of ART is a crucial matter for both medical practitioners and couples who are looking for options to treat infertility, and no part of the treatment process should be overlooked. Some studies have reported that the outcome of ART treatment may depend on the psychological factors of the patient, such as the level of pain before and during ART treatment (11, 12). It is widely accepted that stress, depression, and other psychological disorders hurt pregnancy outcomes (8, 12). However, despite years of research on psychological factors and IVF outcomes, results remain conflicting. We found that most studies on the impact of depression on assisted reproductive outcomes in infertile women focus on clinical pregnancy rates, IVF live birth rates, and miscarriage rates. The purpose of this study is to investigate the correlation between depression in infertile women and the number of oocytes and embryos during the IVF treatment cycle.

## 2 Methods

### 2.1 Participants and study design

**Figure 1:**
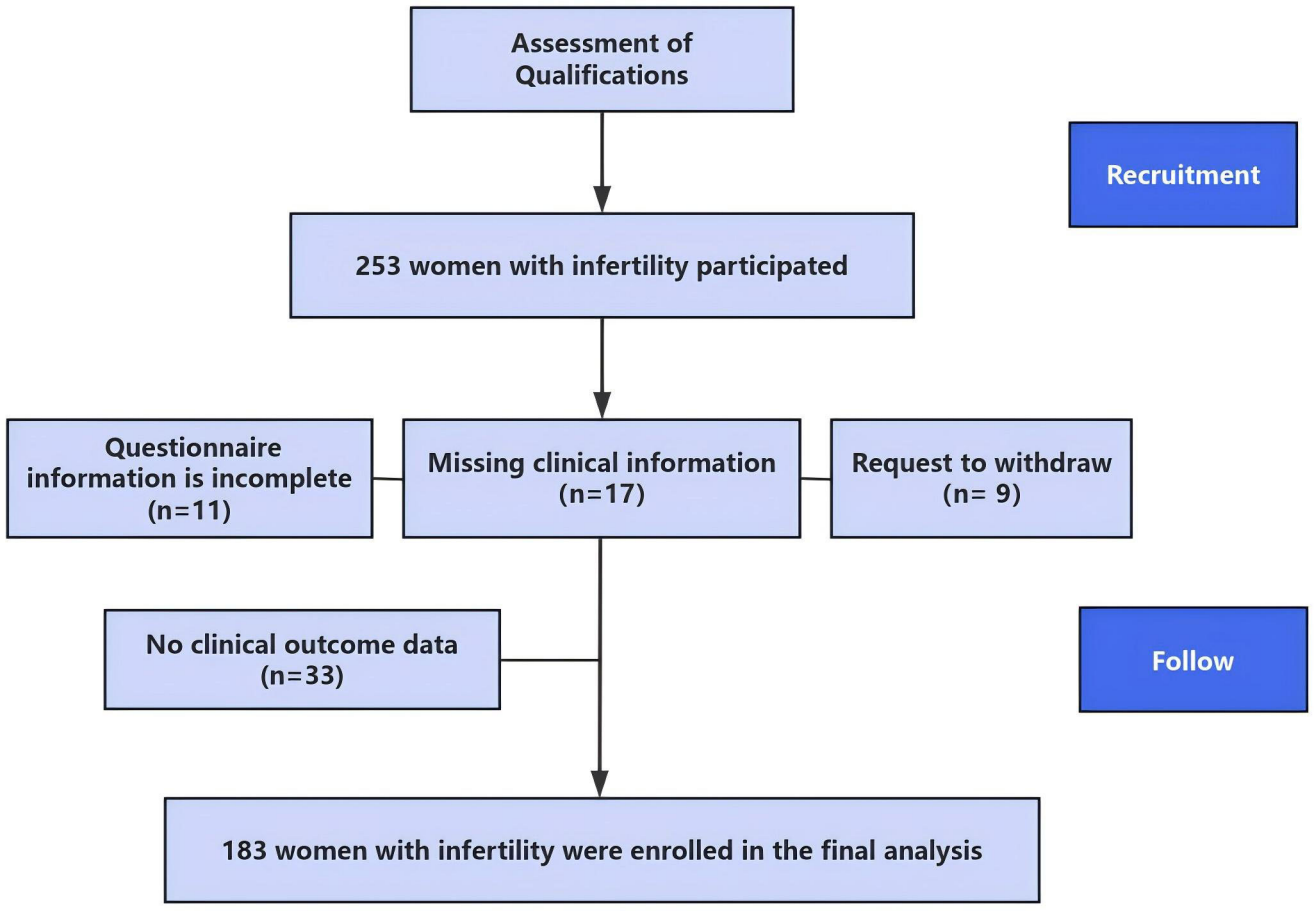
Flowchart of the participant recruitment.

This prospective cohort study was conducted at the Reproductive Medicine Center of Anhui Medical University Affiliated Hospital. In July 2021, participants were recruited using a consecutive sampling strategy from all eligible patients attending the reproductive medicine center. Based on predefined inclusion and exclusion criteria, a total of 253 women with infertility were enrolled. The inclusion criteria were 1. diagnosed with infertility, 2. aged over 18 years old, and 3. confirmed to be receiving IVF-ET treatment. Women who were unable to use mobile electronic devices, had limited comprehension ability, or were deemed unsuitable for IVF-ET were excluded. Follow-up tracking was conducted in November, and clinical outcome data were collected through the electronic medical record system. A total of 183 participants provided complete questionnaire responses and clinical data and were included in the final analysis. **Figure 1** shows the participant recruitment flowchart. The Anhui Medical University Ethics Committee approved and permitted the conduct of this study (approval number 20200961), and all patients gave informed consent.

### 2.2 Depression measures

Depressive symptoms were assessed before the initiation of the IVF cycle using the Patient Health Questionnaire-9 (PHQ-9), which was completed independently by participants. The PHQ-9 is a widely validated instrument with strong reliability and validity across diverse populations (13). The PHQ-9’s nine items align with the diagnostic and statistical manual of mental disorders (DSM) criteria, exhibiting robust screening and diagnostic properties. Each item is structured with four categorical options, coded as follows: not at all = 0, a few days = 1, more than half the time = 2, and almost every day = 3. The total score is the simple sum of the item scores, with cutoffs of 5, 10, 15, and 20 representing thresholds for mild, moderate, severe, and very severe depressive symptoms, respectively (14). In this study, we divided participants into a depression group and a non-depression group. Participants with a PHQ-9 score of 10 or higher were considered to have clinically significant depressive symptoms, while those with a score below 10 were classified as non-depressed. PHQ-9 scores were analyzed both as a categorical variable (≥10 vs <10) and as a continuous variable in spline analyses.

### 2.3 Reproductive outcomes

Clinical outcome data were extracted from electronic medical records after completion of the IVF cycle. Controlled ovarian stimulation was performed using individualized protocols based on patients’ clinical characteristics, including gonadotropin-releasing hormone (GnRH) agonist or antagonist regimens. Ovarian stimulation was followed by transvaginal ultrasound-guided oocyte retrieval for *in vitro* fertilization and culture (15). Retrieved oocytes were assessed for maturity and classified as germinal vesicle (GV), metaphase I (MI), metaphase II (MII), or degenerated. After fertilization, the number of fertilized zygotes with two pronuclei (2PN) was recorded in each cycle (16). Embryos graded I–III on day 2 or day 3 of cleavage-stage development were defined as transferable embryos, which could be selected for fresh transfer, cryopreservation, or further culture to the blastocyst stage (17). The primary reproductive outcomes analyzed in this study included the total number of oocytes retrieved, the number of MII oocytes, the number of 2PN zygotes, and the number of transferable embryos.

### 2.4 Statistical analyses

Statistical analyses were performed using SPSS software, version 23.0 (SPSS Inc., Chicago, IL, USA) and R version 4.1.0 (R Development Core Team). Based on data distribution characteristics, continuous variables were expressed as mean ± standard deviation, while categorical variables were presented as frequency and percentage. Differences between groups were assessed using t-tests, chi-square tests, or Fisher’s exact tests, and the association between depressive symptoms and reproductive outcomes was analyzed using multiple linear regression models. The model controlled for confounding factors, including numeric variables age, categorical variables infertility type, income, education level, cause of infertility, family support, initial treatment protocol, baseline follicle-stimulating hormone/luteinizing hormone (FSH/LH) ratio, and first IVF-ET treatment. To evaluate the robustness of the regression estimates given the modest sample size, bootstrap resampling (1,000 iterations) was performed to obtain bias-corrected confidence intervals. Restricted cubic splines (RCS) were used to explore nonlinear associations between depression and reproductive outcomes. All statistical tests were two-sided, with *P* < 0.05 considered statistically significant.

## 3 Results

**Table 1** summarizes the baseline characteristics of the 183 infertile women stratified by depressive symptom status. The mean age of participants was 30.80 ± 4.05 years, with an age range of 21 to 41 years. Among them, 95 women were diagnosed with primary infertility, and 88 with secondary infertility. More than half of the infertile women had an annual income of less than 30,000 yuan and an educational level of high school or below. Overall, 92.3% of participants reported receiving family support for IVF-ET treatment. Female-related factors accounted for 63.9% of infertility etiologies, and 49.7% infertile women underwent antagonist regimen, of whom 161 women were undergoing their first IVF-ET cycle.

**Table 1:** Characteristics of infertile women in depression score categories (*n* = 183).

Characteristic	Total	Non-depression (*n* = 150)	Depression (*n* = 33)	*P-*value ^a^
Age, years, mean ± SD	30.80 ± 4.05	30.74 ± 3.91	31.09 ± 4.69	0.688
Infertility Type, n (%)				0.412
Primary Infertility	95 (51.9)	80 (53.3)	15 (45.5)	
Secondary Infertility	88 (48.1)	70 (46.7)	18 (54.5)	
Income, RMB, n (%)				0.007
≤ 30.000 yuan	92 (50.3)	68 (45.3)	24 (72.7)	
> 30.000 yuan	91 (49.7)	82 (54.7)	9 (27.3)	
Educational level, n (%)				0.104
High school or below	93 (50.8)	72 (48.0)	21 (63.6)	
College degree and above	90 (49.2)	78 (52.0)	12 (36.4)	
Family support, n (%)				0.286
Supports	169 (92.3)	140 (93.3)	29 (87.9)	
Objects	14 (7.7)	10 (6.7)	4 (12.1)	
Causes of infertility, n (%)				0.615
Men	7 (3.8)	7 (4.7)	0 (0.0)	
Female	117 (63.9)	94 (62.7)	23 (69.7)	
Communion of spouses	25 (13.7)	22 (14.7)	3 (9.1)	
Unknown	34 (18.6)	27 (18.0)	7 (21.2)	
Basal FSH/ LH ratio, n (%)				0.468
≤ 1.09	66 (36.1)	48 (32.0)	12 (36.4)	
1.10 - 1.50	33 (18.0)	49 (32.7)	13 (39.4)	
> 1.50	84 (45.9)	53 (35.3)	8 (24.2)	
Initial treatment protocol, n (%)				0.359
Agonist regimen	68 (37.2)	59 (39.3)	9 (27.3)	
Antagonist regimen	91 (49.7)	71 (47.3)	20 (60.6)	
Other	24 (13.1)	20 (13.3)	4 (12.1)	
First IVF-ET treatment, n (%)				0.229
Yes	161 (88.0)	134 (89.3)	27 (81.3)	
No	22 (12.0)	16 (10.7)	6 (18.2)	
No. of total oocytes, mean ± SD	14.17 ± 8.74	14.64 ± 8.79	12.03 ± 8.34	0.121
No. of MII oocytes, mean ± SD	9.21 ± 6.04	9.56 ± 5.97	7.61 ± 6.18	0.092
No. of 2PN zygotes, mean ± SD	8.23 ± 5.78	8.58 ± 5.73	6.64 ± 5.81	0.080
No. of transferable embryos, mean ± SD	5.54 ± 4.27	5.93 ± 4.23	3.76 ± 4.05	0.008

SD: standard deviation; FSH: follicle-stimulating hormone; LH: Luteinizing hormone; IVF-ET: *in vitro* fertilization-embryo transfer; No.: number; MII, metaphase II; 2PN: zygotes presenting with 2 pronuclei.

^a^ : From the t-test for continuous variables and chi-square^2^ tests or Fisher's exact test for categorical variables.

Regarding IVF treatment outcomes, the mean number of oocytes retrieved was 14.17 ± 8.74, the mean number of MII oocytes was 9.21 ± 6.04, the mean number of 2PN zygotes was 8.23 ± 5.78, and the mean number of transferable embryos was 5.54 ± 4.27. Compared with non-depressed women, those with depressive symptoms showed significant differences in income and the number of transferable embryos (*P* < 0.05), whereas no significant differences were observed for other baseline variables (**Table 1**).

**Table 2** presents the associations between depressive symptoms and reproductive outcomes based on multivariable linear regression analyses. In the unadjusted model, higher depressive symptom scores were significantly associated with a lower number of transferable embryos (β = −2.17, *P* = 0.007). After adjustment for age, infertility type, income, educational level, family support, causes of infertility, basal FSH/LH ratio, initial treatment protocol, and first IVF-ET treatment, depressive symptoms remained significantly associated with a reduced number of transferable embryos (β = −2.17, *P* = 0.004). In addition, the association between depressive symptoms and total oocyte number reached borderline statistical significance in the adjusted model (β = −3.02, *P* = 0.049). No statistically significant associations were observed for other reproductive outcomes. Overall, after adjustment for potential confounders, depressive symptoms were significantly associated with a reduced number of transferable embryos, while associations with total oocytes were borderline, and those with MII oocytes and 2PN zygotes were not statistically significant.

**Table 2:** Relationship between depression scores and the number of oocytes and embryos in 183 women with *in vitro* fertilization cycles.

	Non-depression	Depression	*P-*value
	β (95 % CI)	β (95 % CI)	
Model 1			
No. of total oocytes	ref	-2.61 (-5.87, 0.66)	0.117
No. of MII oocytes	ref	-1.96 (-4.21, 0.30)	0.089
No. of 2PN zygotes	ref	-1.94 (-4.09, 0.21)	0.077
No. of transplantable embryos	ref	-2.17 (-3.74, -0.60)	0.007
Model 2			
No. of total oocytes	ref	-3.02 (-6.05, -0.002)	0.049
No. of MII oocytes	ref	-1.89 (-3.99, 0.22)	0.079
No. of 2PN zygotes	ref	-1.77 (-3.80, 0.27)	0.088
No. of transplantable embryos	ref	-2.17 (-3.64, -0.69)	0.004

No: number; MII, metaphase II; 2PN, zygotes presenting with 2 pronuclei; β: regression coefficient; CI, confidence interval; Model 1, Simple Analysis, Model 2 adjusted for age, infertility type, income, educational level, family support, causes of infertility, basal FSH/ LH ratio, initial treatment protocol and first IVF-ET treatment.

**Figure 2:**
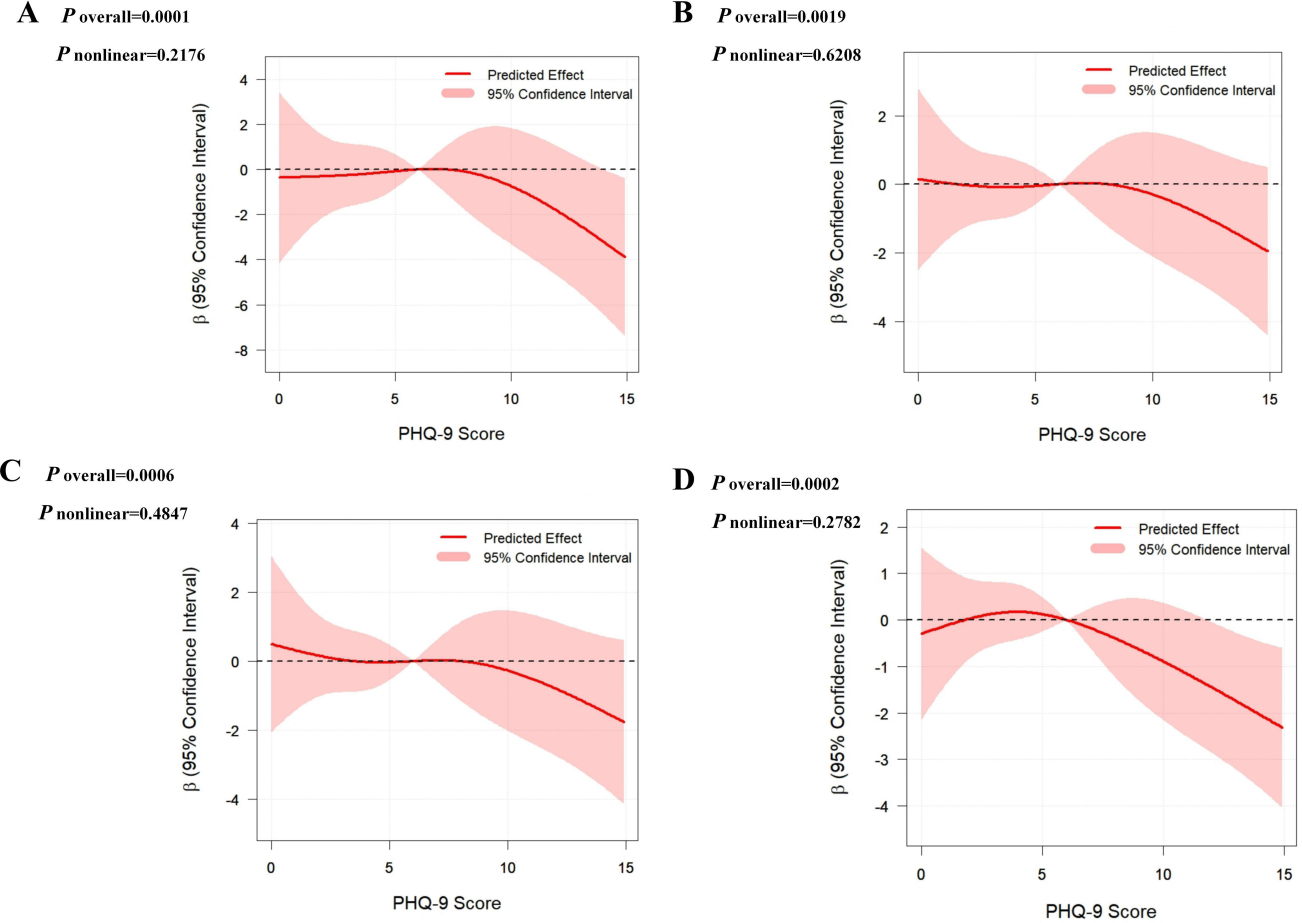
Dose–response associations between depressive symptoms and IVF outcomes. Restricted cubic spline models show the associations between PHQ-9 score and **(A)** total number of oocytes, **(B)** number of metaphase II (MII) oocytes, **(C)** number of two-pronuclear (2PN) zygotes, and **(D)** number of transferable embryos. All models were adjusted for confounding factors.

The bootstrap standard errors and 95% CIs were highly consistent with the original estimates, with minimal bias estimates and overlapping confidence intervals. (**Supplementary Table 1**). The research findings showed a high degree of stability. Furthermore, as shown in **Figure 2**, the RCS model indicates that as PHQ-9 scores gradually increase, the regression coefficients β for outcome measure show an overall downward trend. Specifically, for total oocytes retrieved (A), MII oocytes (B), 2PN zygotes (C), and transferable embryos (D), the overall dose-response relationship between PHQ-9 scores and outcomes was statistically significant (*P* overall < 0.01 for all), while nonlinear tests failed to reach statistical significance (*P* nonlinear > 0.05).

## 4 Discussion

Women with infertility are at increased risk of depressive symptoms due to the psychological burden of complex treatment procedures, financial stress, diminished quality of life during IVF-ET, and broader social and emotional pressures (18, 19). Previous research by Namdar et al. reported that more than half of infertile women experienced some degree of impaired general health and were at elevated risk of anxiety, social dysfunction, and depression (4). In the present study, 18.1% of infertile women met the criteria for moderate depressive symptoms, a prevalence comparable to that reported in earlier studies (20, 21). Given the growing number of women undergoing assisted reproductive treatments, these findings underscore the importance of recognizing and addressing depressive symptoms as part of comprehensive infertility care. The present study specifically focuses on intermediate reproductive indicators during IVF treatment, aiming to better understand factors associated with earlier stages of reproductive development before implantation and pregnancy outcomes.

Previous studies have consistently demonstrated that obtaining an adequate number of mature oocytes is critical for the success of ART, as a higher oocyte yield increases the likelihood of generating high-quality embryos and achieving live birth (22–24). Accordingly, the present study focused on intermediate outcomes during IVF-ET treatment, particularly the number of oocytes retrieved and the number of transferable embryos. As IVF-ET progresses, the number of embryos typically decreases due to attrition during fertilization and early embryonic development, highlighting the importance of sufficient oocyte yield at the initiation of treatment. In this context, the number of transferable embryos—defined as embryos suitable for fresh transfer, cryopreservation, or further culture to the blastocyst stage—represents a key determinant of cumulative pregnancy potential. Importantly, although intermediate outcomes such as the number of transferable embryos reflect ovarian response and cumulative reproductive potential, they do not directly equate to ultimate reproductive success (25). Clinical pregnancy and live birth depend on multiple additional factors, including embryo implantation, endometrial receptivity, and maternal physiological conditions (26). Therefore, the findings of the present study should be interpreted as reflecting associations at an earlier stage of the IVF process rather than definitive predictors of final treatment outcomes. Given the complex, time-consuming, and costly nature of ART, as well as the possibility of fertilization failure, embryonic arrest, or unsuccessful transfer, the availability of multiple transferable embryos is often necessary to support repeated transfer attempts (27). Notably, previous studies have shown comparable live birth, implantation, and clinical pregnancy rates between frozen and fresh embryo transfers, further emphasizing the clinical value of having a sufficient pool of transferable embryos (28, 29). A successful IVF-ET cycle requires the presence of at least one transferable embryo, adequate endometrial receptivity, and effective synchronization between embryo development and the uterine environment. Within the biopsychosocial theoretical framework, depressive symptoms may be associated with stress-related physiological responses, which affect reproductive function through neuroendocrine pathways. Chronic psychological stress has been shown to suppress hypothalamic–pituitary–ovarian axis activity by inhibiting gonadotropin-releasing hormone secretion, potentially impairing ovarian function, oocyte quality, and subsequent embryo development (30). These mechanisms may partially explain the observed association between depressive symptoms and reduced embryo availability in the present study.

Existing evidence regarding the impact of depression, anxiety, and other mood disorders on ART outcomes remains inconclusive. While many studies have reported that psychological distress is associated with poorer ART outcomes, including reduced success, live birth, and full-term pregnancy rates (8, 11), others have found no significant associations between mental health disorders and assisted reproduction outcomes (20, 31). Notably, most of these studies have focused primarily on final clinical endpoints, such as pregnancy or live birth rates. In contrast, the present study examined intermediate reproductive outcomes during IVF-ET cycles, providing additional insight into earlier stages of the reproductive process.

Our findings suggest that depressive symptoms are associated with a reduced number of transferable embryos, which may partially explain previously observed associations between psychological distress and ART success (20, 31). Consistent with this interpretation, Zhou et al. reported that adverse psychological states can disrupt immune homeostasis at the maternal–fetal interface, thereby impairing embryo transfer success in IVF-ET (32, 33). These findings highlight the potential value of integrating psychological assessment and supportive interventions throughout the IVF-ET treatment process. Such a comprehensive approach may help alleviate psychological distress and indirectly contribute to improved reproductive outcomes. These findings suggest that psychological screening before IVF initiation may help identify patients at risk of reduced embryo availability, potentially allowing early supportive interventions.

Several limitations of this study should be acknowledged. First, the sample size was relatively modest and included only female participants, precluding evaluation of couple-level psychological interactions. *Post hoc* power considerations suggest limited power for detecting small effect sizes. Second, depressive symptoms were assessed only at the early stage of IVF-ET, and changes in psychological status throughout treatment were not captured. In addition, the PHQ-9 scale used is not an objective criterion for the clinical diagnosis of depression. Future studies with larger samples, inclusion of both partners, and longitudinal assessments of psychological well-being are warranted to further clarify these associations.

## 5 Conclusion

Women undergoing IVF-ET treatment for infertility exhibit a higher incidence of depression, depressive symptoms were associated with less favorable intermediate reproductive outcomes during the assisted reproductive process, especially the number of transplanted embryos. To enhance assisted reproductive success rates, the entire treatment process should be considered when evaluating pregnancy outcomes.

## Data Availability

The original contributions presented in the study are included in the article/**Supplementary Material**. Further inquiries can be directed to the corresponding authors.

## Supplementary material

Supplementary Table 1 Bootstrap analysis. Note: No: number; MII, metaphase II; 2PN, zygotes presenting with 2 pronuclei; β: regression coefficient; CI, confidence interval; Estimating the robustness of the effect using the Bootstrap self-sampling method (with 1,000 repetitions).
