# Supplementary material for: Psychological depression and its association with oocyte yield and embryo outcomes in infertile women undergoing in vitro fertilization-embryo transfer
Source: Front Psychiatry. 2026 May 15;17:1796497. doi: 10.3389/fpsyt.2026.1796497 (PMC13219258; doi:10.3389/fpsyt.2026.1796497)
Supplement: Supplementary Table 1 — Bootstrap analysis. Note: No: number; MII, metaphase II; 2PN, zygotes presenting with 2 pronuclei; β: regression coefficient; CI, confidence interval; Estimating the robustness of the effect using the Bootstrap self-sampling method (with 1,000 repetitions). [file Table1.docx]

**Supplementary Table 1 Bootstrap Analysis**

| Outcomes | Original (β) | Bias | Std. Error | 95% CI |
| --- | --- | --- | --- | --- |
| No. of total oocytes | -3.02 | 0.023 | 1.701 | (−6.35, 0.31) |
| No. of MII oocytes | -1.89 | 0.016 | 1.190 | (−4.22, 0.44) |
| No. of 2PN zygotes | -1.77 | 0.024 | 1.119 | (−3.96, 0.42) |
| No. of transplantable embryos | -2.17 | 0.014 | 0.761 | (−3.66, −0.68) |

Note: No: number; MII, metaphase II; 2PN, zygotes presenting with 2 pronuclei; β: regression coefficient; CI, confidence interval; Estimating the robustness of the effect using the Bootstrap self-sampling method (with 1,000 repetitions).
